# Nascent Inquiry, Metacognitive, and Self-Regulation Capabilities Among Preschoolers During Scientific Exploration

**DOI:** 10.3389/fpsyg.2020.01790

**Published:** 2020-07-21

**Authors:** Ronit Fridman, Sigal Eden, Ornit Spektor-Levy

**Affiliations:** ^1^Faculty of Social Sciences, School of Education, Bar-Ilan University, Ramat Gan, Israel; ^2^The School of Education, Bar Ilan University, Ramat Gan, Israel

**Keywords:** early childhood, science education, inquiry, metacognition, self-regulation, exploration

## Abstract

There is common agreement that preschool-level science education affects children’s curiosity, their positive approach toward science, and their desire to engage with the subject. Children’s natural curiosity drives them to engage enthusiastically in all forms of exploration. Engaging in scientific exploration necessitates self-regulation capabilities and a wide repertoire of cognitive and metacognitive strategies. The purpose of this study was to examine to what extent preschoolers (aged 5–6 years) implement nascent inquiry skills, metacognitive awareness, and self-regulation capabilities during play-based scientific exploration tasks. An additional purpose was to investigate the relationships between these capabilities, a relationship not yet investigated in the context of play-based, scientific exploration among young children. The study consisted of 215 preschoolers, from 10 preschools. For this study, we developed two scientific exploration tasks – structured and open-ended. Our motivation was to examine whether preschoolers’ capabilities will differ in the context of structured task which is aligned with the view that young children need guidance and explicit instructions compared to the context of open-ended, play-based task–allowing the children to apply and test their intuitive theories and skills. During performance participants were videotaped. Their verbal and non-verbal responses were analyzed by means of a coding scheme. The results of a micro-analysis of about 100 h of video showed that given the opportunity, even without setting explicit goals and instructions, children exhibit inquiry capabilities: they ask questions, plan, hypothesize, use tools, draw conclusions. Asking questions and planning were better manifested during the structured task. Children also manifested higher levels of attention, persistence, and autonomy during the structured task. However, significant higher scores of self-regulation indications were revealed in the context of the open-ended, play-based, exploration task. Moreover, results indicate significant correlations between the five measures of preschoolers’ inquiry capabilities and measures of metacognitive strategic awareness and self-regulation. The results of the present study suggest the importance of combining various learning environments and experiences in early science education that encourage children to engage in structured exploration alongside play-based, open-ended, exploration.

## Introduction

There is a consensus among researchers that preschool-level science education affects children’s curiosity, their positive approach toward science, and their desire to engage with the subject (Eshach, 2006; Patrick et al., 2009; Worth, 2019). These factors predict the likelihood of engaging in science and of scientific achievements in both the short and long terms (Osborne et al., 2003; Tao et al., 2012). The American National Science Teachers Association (National Science Teachers Association [NSTA], 2014) recommended the adoption of an official position on teaching science in early childhood, claiming that the scientific inquiry process is based on principles of active and independent activity on the part of the child. The aspiration is to bring the child to a level of open, independent inquiry, wherein s/he can raise research queries on her/his own, plan the research, and carry it out. In addition, the National Science Teachers Association [NSTA] (2014) presented principles aimed at shedding light on those activities, and ways to make engagement in science accessible to children to help them develop skills and knowledge over time. As engagement in science is a knowledge-building process, it is recommended to expose children to many varied opportunities to engage in scientific inquiry processes on a regular basis. Children need time to play, to observe new phenomena, to think about what they have seen and discovered, and to draw conclusions (National Science Teachers Association [NSTA], 2014). These complex capabilities require learners to criticize and regulate their own thinking, learning, and outcomes. Thus, the scope of this study involves identifying these nascent capabilities in order to nurture and develop them through providing inquiry-based learning opportunities.

## Literature Review

### Early Childhood Science Education

Over the years, scholarly literature has increasingly acknowledged the advantages of beginning science education in early childhood (Eshach, 2003; National Science Teachers Association [NSTA], 2014; Early Childhood Stem Working Group, 2017; McClure et al., 2017).

One important objective of science education is to assist children in discovering the world and to answer their questions as they employ their cognitive and physical skills (Jones et al., 2008). It is essential to present science to children in developmentally appropriate ways, thus enabling them to explore the world through sensory investigations. This also helps children to absorb basic knowledge and abilities that are necessary for lifelong science learning and appreciation of nature (Trundle and Saçkes, 2015). The guided process could begin by observing children during their explorations and offering them appropriate support to improve their thinking and inquiries.

Some researchers view the child as a natural scientist (Gopnik, 2012), a contention based on studies indicating that young children possess cognitive abilities enabling them to comprehend scientific concepts and implement inquiry skills (Buchsbaum et al., 2011). According to this view, children understand and explain the world by means of intuitive theories and are able to transform these theories in light of new, cumulative knowledge (Williams and Lombrozo, 2013). In contrast, other studies argue that children have difficulty spontaneously acquiring the components of scientific inquiry and implementing scientific thinking skills – such as designing experiments, recording data, and processing existing knowledge with new knowledge – without guidance and explicit instruction. Therefore, they must be taught all these capabilities in a structured way (Zimmerman, 2007).

### Inquiry in Preschool

Implementation of early childhood science curricula and exposing children to enjoyable, exciting science activities can help them develop scientific knowledge, inquiry capabilities, scientific discourse, and positive attitudes toward science (French, 2004; Samarapungavan et al., 2008; Patrick et al., 2009). Policy papers and scholarly literature have recommended teaching science in the manner conducted by scientists; that is, via inquiry (e.g., National Research Council (NRC), 2012; NGSS Lead States, 2013). Inquiry-based learning reflects the constructivist approach, according to which educators must strive to create a learning environment wherein the learners are required to examine thought processes: to gather, record, and analyze data; to analyze and test hypotheses; to test prior knowledge, and to formulate new significance on their own (Dewey, 1938; Kuhn et al., 2000; Greeno, 2002; Loyens and Rikers, 2011; Andiema, 2016). Indeed, young children can hypothesize and modify hypotheses as needed (Legare, 2012); provide explanations that draw upon high-level, abstract thinking; and process existing knowledge with new knowledge (Schulz, 2012; Williams and Lombrozo, 2013). They can carry out observations, establish hypotheses based on evidence, understand experiments, identify reliable information sources (Klahr et al., 2011), use their own hypotheses to predict results, and evaluate evidence (Piekny and Maehler, 2013; Walker et al., 2014).

It is recommended that children be engaged in science topics, and that science constantly be incorporated into other subjects learned in preschool (National Research Council (NRC), 2012; NGSS Lead States, 2013). The first step in teaching the sciences is to enable children to engage with various objects and materials and to provide a range of possibilities, materials, and opportunities (Sheridan and Samuelsson, 2001). It is fundamental to direct any activity with suitable inquiries and practices, rather than tell the children what to do (Tunnicliffe, 2015). The engagement with scientific thinking skills in such varied ways provides children with opportunities to develop other skills, such as mathematical language and social skills (Bustamante et al., 2018). Moreover, science learning and the scientific inquiry process provide stimulating contexts for the development of metacognitive and self-regulation capabilities (Michalsky et al., 2007; Zimmerman, 2008; Jirout and Zimmerman, 2015).

### Metacognition and Self-Regulation

In recent years, the term *metacognition* has been widely used in the field of education and the study of metacognition has evolved into a flourishing field of research in cognitive psychology. Flavell (1976), who coined the term metacognition, defined it as the individual’s knowledge, regulation, and control of the processes and outcomes of her/his own cognitive system. Over the years, this definition has expanded to include the emergence of associated concepts such as reflection, self-regulation, metacognitive awareness, etc. Metacognition is higher-order thinking, and includes critical thinking about one’s own thinking, planning, controlling, monitoring, assessing, knowing what information is required for a task, and how to use the appropriate tools needed to perform the task (Flavell, 1979).

The increased number of studies of the concept of metacognition has led to confusion and discussions about the distinctions between the various components of metacognition and their relationships (Veenman et al., 2006). The scope of this review does not allow for a survey of all the approaches and taxonomies of metacognition.

We have adopted the view that distinguishes between two major components of metacognition: knowledge about cognition and regulation of cognition (Flavell, 1976; Brown, 1987; Schraw and Dennison, 1994). Knowledge about cognition is realized through three reflective processes: declarative knowledge (what), procedural knowledge (how), and conditional knowledge (when and why). Regulation of cognition is realized through the following processes: planning, monitoring, and management of strategies – control, debugging, and evaluation (Schraw and Dennison, 1994; Schraw and Moshman, 1995).

In this study, regulation of cognition is called *self-regulation*. Self-regulation refers to how we use external and internal clues to determine when to start, continue, or stop a particular action to achieve the desired goal. Accordingly, self-regulation includes the ability to choose behaviors while controlling the intensity of the response, the ability to plan the response, and to respond effectively during internal and interpersonal discourse (Zimmerman, 2000).

Self-regulation is one indicator of the relationship between emotion and cognition (Lee, 2005). A wide range of motivation-related cognitive interactions and metacognition control the process of acquiring self-regulation skills (Zimmerman, 2000; Schraw et al., 2006). Motivation relates to perceptions, attitudes, and desires that influence the use and development of cognitive and metacognitive skills (Schraw et al., 2006).

At every age, self-regulation for learning is the ability to identify or set goals; identify a mismatch between goals and the state of one’s current expertise; monitor learning behaviors continuously and accurately; and initiate regulation processes for the performance of a task (Best and Miller, 2010).

### Metacognition and Self-Regulation Among Young Children

According to Vygotsky (1978, 1986), learning begins in a social context, where an adult supports the young learner, providing her/him with a safety net. It is a process of internalization that begins with regulation through others and evolves into self-regulation.

Evidence of self-regulation of learning and cognition in early childhood has accumulated in recent decades (e.g., Istomina, 1975; Schweinhart and Weikart, 1997; Bronson, 2000; Whitebread et al., 2005, 2007, 2009; Blair and Razza, 2007; Rodríguez and Palacios, 2007; Hattie, 2009). In addition, much research evidence about metacognitive knowledge and self-regulation has accumulated regarding children of 3–5 years of age (Mevarech, 1995; Shamir et al., 2009; Whitebread et al., 2009).

Studies such as the longitudinal national cohort study, Pre-COOL (Mulder et al., 2014), and the Effective Provision of Pre-School Education (EPPE) project (Sylva et al., 2004) have indicated the importance of self-regulated learning in early childhood. Fostering self-regulated learning as early as possible is crucially important, since children develop their learning abilities during their first early years (De Corte et al., 2000; Hendy and Whitebread, 2000). Bryce and Whitebread (2012), as well as Bryce et al. (2015), contend that monitoring and control are some of the abilities that have already been developed by preschool age. Therefore, it seems prudent to foster young children’s metacognitive and self-regulation capabilities at an early stage (Winne and Hadwin, 2008). Fostering these capacities proves beneficial to scholastic performance (Butler et al., 2004; Blair and Razza, 2007; Hidi and Ainley, 2008; Rimm-Kaufman et al., 2009; Moffitt et al., 2011; Butler and Schnellert, 2012; Diamond et al., 2013; Dunn et al., 2014; Kitsantas et al., 2017; Dörr and Perels, 2019).

Metacognitive thinking develops intuitively in children, along with the evolution of intelligence through their interaction with the environment, with parents, with teachers, with friends, and with others (Beishuizen and Veenman, 2004). According to Bronson (2000), the optimal environment for encouraging the development of emotional and behavioral self-regulation has a number of characteristics: It is regular, safe, sufficiently stimulating, responsive, and sensitive to the child’s needs and perspectives, and affords security and encouragement. It sets clear boundaries and standards, offer examples and role modeling, and provides opportunities for activities that are directed toward the development of autonomous self-regulation (Klein and Yablon, 2008).

In this study we will focus on nascent metacognitive thinking in the context of play-based scientific experiments among young children. Since it is challenging to differentiate between the three components comprising knowledge of cognition: (declarative, procedural, and conditional; Schraw and Dennison, 1994) in very young children, we will relate to the general manifestation of metacognitive strategic awareness (e.g., looking for evidence like planning, looking at the available materials, and pausing to think). We will also look for evidence of nascent self-regulation (e.g., task awareness, planning, monitoring and debugging, controlling, and evaluating).

### Inquiry and Self-Regulation Capabilities

Over the past three decades, research on cognitive development has yielded data on the development of children as independent learners. Inquiry and exploration processes provide opportunities to develop independent learning capabilities. During the course of inquiry processes, a process of formulating knowledge and, when needed, transforming knowledge takes place, and more sophisticated inquiry skills are developed. Concurrently, as metacognitive and meta-strategic knowledge develop, children and adolescents gain a better understanding of the nature of inquiry and the skills used in the inquiry process. Thus, the entire process is iterative and cyclical, involving some or all of the components of scientific inquiry – such as designing experiments, evaluating evidence, and drawing inferences – serving to form and/or revise theories about the phenomena under investigation (Jirout and Zimmerman, 2015). Kuhn (1989, 2002) contends that the defining feature of scientific thinking is the set of cognitive and metacognitive skills involved in differentiating and coordinating theory and evidence. In particular, metacognitive awareness is what differentiates more sophisticated scientific thinking from less sophisticated. Instruction that develops higher-order thinking contributes to building students’ knowledge and aids their transition from memorizing and rote learning to learning that emphasizes the building of knowledge in meaningful ways. It encourages their self-regulation and motivation to succeed at science by means of modeling effective inquiry strategies (Chinn and Malhotra, 2002). Therefore, understanding the ways nascent inquiry and self-regulation capabilities emerge, develop and are mutually related in early childhood is a prerequisite.

A review of the literature on early childhood science education reveals that despite the existing volume of research accumulated over the years, what we know about very young children’s science learning is limited, as most of the research has focused on the later elementary school years and beyond (Trundle and Saçkes, 2015). While this may be partially due to a lack of priority in early childhood research funding, it is also important to acknowledge the challenges of researching scientific knowledge and understanding among very young children, where language, both oral and written, may not reflect children’s skills, reasoning, and understanding (National Science Teachers Association [NSTA], 2014). Therefore, the main aim of this study is to analyze verbal and non-verbal responses of preschool children (5–6 years of age), to identify their nascent and intuitive inquiry thinking and behaviors, metacognitive strategic awareness, self-regulation capabilities and the relation between these capabilities, while they are engaged in structured and open-ended, play-based scientific experiences. Previous studies, as noted above, showed significant evidence that engaging in scientific inquiry processes necessitates self-regulation capabilities and a wide repertoire of cognitive and metacognitive strategic awareness. The extent to which these combined capabilities are developed and manifested in early childhood are rarely investigated among preschool children (5–6 years of age), particularly in the context of open-ended scientific exploration.

### Play-Based Scientific Exploration

A great deal of research has focused on testing and describing children’s responses while encountering a novel occurrence in a structured setting and inferring the children’s assumptions and conceptions. Less attention has been paid to the ways that these processes occur in everyday environments and the ways these processes occur in free-play and play-like situations (Wager and Parks, 2014). Why play? Researchers and educators are often skeptical of play-based learning activities, seeing them as just play. However, as Bergen (2009) contends, playfulness appears to provide a predisposition toward certain types of creative acts, including those employed in scientific and mathematical fields. Play is valuable for children primarily because it is a medium for development and learning (Bergen, 2009). Play enables children to examine materials and try out techniques in artistic and creative endeavors. Therefore, scientific exploration tasks may provide play-based situations in which to examine preschoolers’ intuitive inquiry and self-regulation processes.

In this present study, we designed a mini Manipulative Environment (ME; Bumbacher et al., 2018) that invites preschoolers to explore and manipulate materials using an inquiry-based approach. The representational aspects of the ME relate to the concept of affordances: the information a ME provides which builds the basis for decisions of how to interact with it.

While planning the ME, we were inspired by Fleer (2009) who described a “potion center” (p. 1076) to explore materials and their properties. The potion-play involved the provision of numerous plastic bottles, plastic tubing, buckets, colored water, and funnels. In addition, at appointed times the children were also given fragrant oils, vinegar, peanut oil, and more. Following Fleer (2009), we designed two mini MEs for play-based scientific exploration implementing both open-ended and structured play-like tasks.

The play-based scientific exploration tasks developed for this study were performed in preschool classrooms. The open-ended tasks did not impose clear task goals for the child to reach. This authentic, ecological methodology has rarely been applied so far, and thus has the potential to contribute to the theoretical and practical knowledge in the field of early science education.

## Research Questions

The purpose of this study is to examine to what extent preschoolers implement nascent intuitive inquiry processes, metacognitive strategic awareness, and self-regulation capabilities during open-ended and structured play-based science experiences, and to what extent a relationship exists between these capabilities. Such relationships have not yet been studied among young children in the context of an authentic, ecological approach involving play-based science experiences in the preschool classroom.

We gathered data directly from the children during the performance of two scientific exploration tasks. Our three research questions and hypotheses were as follows:

1. To what extent do preschoolers manifest intuitive inquiry capabilities during open-ended and structured play-based scientific exploration tasks?

In this present study, we hypothesize that in the context of play, children will perform intuitive inquiry processes to a significant extent. This hypothesis is based on structured experiments showing that young children can carry out observations, establish hypotheses based on evidence, interpret, and infer (Klahr et al., 2011; Piekny and Maehler, 2013; Walker et al., 2014). Moreover, Bergen (2009) argues that free play is valuable for children and provides an enjoyable medium for development and learning. Play enables children to examine materials and try techniques in explorative and creative endeavors. Therefore, our hypothesis will be tested in an open-ended, play-based scientific exploration task as well as in a structured task.

2. To what extent do preschoolers manifest metacognitive strategic awareness and self-regulation capabilities during open-ended and structured play-based scientific exploration tasks?

We hypothesize that in the context of play, preschoolers will manifest similar significant metacognitive strategic awareness and self-regulation capabilities as previously noted in the Literature Review, whereby there is accumulated evidence of preschoolers’ capabilities to perform metacognitive awareness and self-regulation capabilities in the context of structured tests (Bronson, 2000). In a previous study, young children showed such capabilities during play-based construction tasks (Spektor-Levy et al., 2017). Therefore, our hypothesis will be tested in an open-ended, play-based scientific exploration task as well as in a structured task.

3. What is the nature of the relationship between the implementation of inquiry processes, metacognitive strategic awareness, and self-regulation capabilities among preschoolers during an open-ended and structured play-based scientific exploration task?

We hypothesize that positive, medium-strength correlations will be found between the implementation of inquiry processes, metacognitive awareness, and self-regulation capabilities among preschoolers during an open-ended and structured play-based scientific exploration task, as the literature is replete with such evidence among older students (Kuhn, 1989, 2002; Jirout and Zimmerman, 2015). Following a thorough search of the scholarly literature, we scarcely found any studies that examined the relations between metacognitive strategic awareness, self-regulation capabilities and inquiry capabilities among preschoolers during authentic play-based scientific exploration in the context of scientific exploration tasks.

## Methodology

### Study Sample

The study sample consisted of 215 children: 120 boys (55.8%) and 95 girls (44.2%). The mean age of the participants was 64.79 months (*SD* = 4.2). Children were randomly assigned from 10 urban, middle SES, mainstream preschool classrooms, with parents’ consent. The study was reviewed and approved by the Ethics Committee #10495, The Office of the Chief Scientist of the Ministry of Education, Israel.

Baseline measures were collected using two tools: The *Raven’s Progressive Matrices* (Raven, 1956), designed to measure general cognitive ability and meaning-making. Raven scores ranged from 85 to 129 (*M* = 110.07; *SD* = 10.75). The *Peabody Picture Vocabulary Test* (PPVT-4; Dunn and Dunn, 2007), which measures an individual’s receptive vocabulary, providing an estimate of verbal ability and scholastic aptitude. PPVT scores ranged from 85 to 128 (*M* = 109.43; *SD* = 8.38). All participants scored within the typical range. None of the participants were diagnosed as having any developmental or language delays, nor any motor disabilities.

In Israel, the preschool (3–6 years of age) curriculum requires the implementation of an obligatory Science and Technology curriculum that emphasizes inquiry activities. However, all teachers of the 10 preschool classrooms in this study reported that they employ neither open inquiry processes nor the acquisition of inquiry practices in their classrooms.

Data collection was carried out over a 2-year period. During the first year, data were collected from 68 children who engaged in the free, open-ended scientific exploration task. During the second year, data were collected from 147 children who engaged in the open-ended scientific task and another task, a structured scientific exploration task. For the entire sample of 215 preschoolers, only descriptive statistics and correlations were used.

The optimal overall sample size was determined a *priori* using G Power software. The desired sample size is at least 197 subjects for a two-tailed hypothesis, a small effect size of 0.25, an alpha 0.05 error, and a very high power of 0.95.

The optimal sample size of the 147 subjects, for whom Wilcoxon tests were performed to analyze paired comparisons, was also determined *a priori* using G Power software. The desired sample size is at least 146 subjects for a two-tailed hypothesis, a small effect size of 0.25, an alpha 0.05 error, and a high power of 0.85.

### Research Procedure

Data collection employed a mixed-method approach, combining qualitative and quantitative research tools. In a mixed method study, the researcher or group of researchers combine elements from the qualitative and quantitative research methods in order to expand, deepen, and reinforce the intellectual and practical insights based on quantitative and qualitative evidence (Johnson et al., 2007).

Task performance was carried out with each child individually, during school hours, in a quiet space within the preschool facility. Each task included a pre- and post-exploration interview. The first phase of the pre-exploration interview was intended to facilitate the acquaintance between the researcher and the child, and to gather background information (e.g., “What’s your name?,” “How old are you?”). The next phase of the pre-exploration interview included the following questions: “Do you like to investigate things? If you do like to investigate things, how do you go about it?,” “What’s on the tray?,” “What would you like to do with the materials and equipment on the tray?,” “What do you think will happen?,” “Do you have any questions? Would you like to know more about anything that’s here?” After the exploration task, a post-interview was conducted: “What happened during your investigation?,” “Why do you think it happened? Can you explain?,” “Are you satisfied/happy with what happened? Why?,” “If you had more time, what else would you have done?,” “Do you like to investigate things like what we did here?”. Next, the two scientific exploration tasks were performed in a counterbalanced manner.

The scientific exploration tasks afforded opportunities for planning, hypothesizing, concluding, asking questions, using scientific tools, self-regulation, and constant awareness of task requirements, monitoring, and problem-solving capabilities involving trial and error.

### Research Tools

#### Exploration Tasks

As young children lack the verbal proficiency necessary for prospective and retrospective self-report measures (e.g., questionnaires, interviews, and thinking aloud), it is increasingly recognized that research with very young children should be based on behavioral, exploratory methodologies (Winne and Perry, 2000; Whitebread et al., 2005). Studies relying less upon children’s verbal abilities have tended to show children to be more knowledgeable and skilled than originally believed (Whitebread et al., 2009; Spektor-Levy et al., 2017). Therefore, for this study, we developed two tasks to identify indications of nascent inquiry, metacognitive strategic awareness, and self-regulation capabilities during play-based scientific exploration.

**FIGURE 1:**
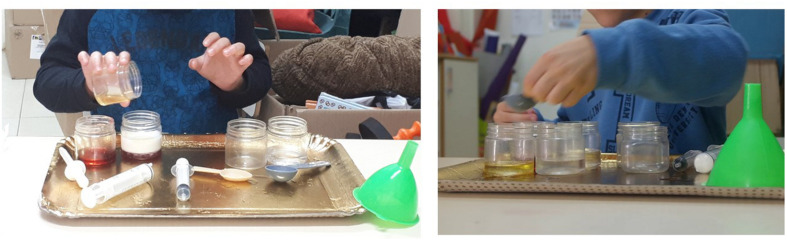
Open-ended and structured scientific exploration tasks.

Both tasks lasted about 15 min and comprised three parts: a pre-exploration interview, a scientific exploration task (open-ended or structured), and a post-exploration interview. In the pre-exploration interview, the participants were asked about their attitudes toward science, what they knew about science, and when and how they had engaged in science. In addition, both interviews (pre- and post-exploration) included questions aimed at examining and identifying the nascent scientific thinking of the children, and their ability to implement components of inquiry such as asking questions, planning, hypothesizing, and drawing conclusions (see Supplementary Data Sheet 1). The interviews and the exploration took place during preschool hours, and each child was interviewed individually spoons (Figure 1).

Throughout the exploration tasks, video-recordings were made of the children’s behavior and responses. Our intention was to closely observe and record the verbal and non-verbal-behavioral responses of the preschoolers while they were intuitively engaged in the exploration tasks. Micro-analysis of the video recordings (described below in the coding scheme) enabled the researchers to quantify the inquiry, metacognitive strategic awareness, and self-regulation capabilities that were identified.

##### Open-ended scientific exploration task (N = 215)

On the table there was a tray with jars containing various liquids (still water, seltzer water, oil, juice, and milk) and the following items: a syringe, two droppers, a funnel, measuring cups, and measuring spoons. The children were asked to experiment freely with the liquids and objects for 5 min. They were told, “You have 5 min to do whatever you want with these items. When the timer goes off, your time is up, and you have to finish.”

##### Structured exploration task (N = 147)

This task followed the Predict-Observe-Explain (POE) approach, which requires that before each experiment learners make predictions about the outcome (Bumbacher et al., 2018). Then they design the experiment and re-examine the predictions in light of their new observations (Rickey and Stacy, 2000). Despite its caveats, POE is a simple intervention that prompts learners to think more carefully about the experiment’s design, expected outcomes, and what can be learned, which manifests intentional and unconfounded experimentation capabilities (Kearney et al., 2001).

In this task, the table held a tray with jars containing liquids (two jars containing still water, one jar containing juice, one containing oil, two empty jars), a syringe, two droppers, a funnel, measuring cups, and measuring spoons (Figure 1). The children were asked to follow these instructions: “Mix half of the water with half of the oil, and half of the water with half of the juice; and then tell us when you’re done. After telling us you’re done, leave the items in place.”

#### Coding Scheme

The coding scheme was based on the Metacognitive Skills in Constructional Play Engagement (MetaSCoPE) coding scheme. It was designed by Bryce and Whitebread (2012) and was further developed for accuracy by Spektor-Levy et al. (2017). For the present study, the coding scheme was adapted to identify emerging inquiry capabilities. The video analysis coded both verbal and non-verbal responses. Non-verbal responses could be manifested by private gestures, that is, signs that children intentionally direct toward themselves or objects (Rodríguez and Palacios, 2007; Basilio and Rodríguez, 2011). Private gestures may reflect a cognitive function (Garber and Goldin-Meadow, 2002; Pine et al., 2004) or manifest as a spontaneous production of gestures when solving tasks that involve the use of spatial information (Chu and Kita, 2008). The analysis also looked for private speech. Private speech emerges during preschool years and becomes critical to the development of self-regulation. It is an intermediate step between self-regulatory external speech, and internal speech (Savina, 2014); and although private speech is spoken aloud, it is used for self-guidance, planning, and problem-solving, rather than for a communicative purpose (Vygotsky, 1997; Lee et al., 2014).

The coding was based on several indicators, which encompassed various indications. For each inquiry indicator, the coding scheme specifies the behaviors and responses that manifest the specific capabilities (Supplementary Data Sheet 1). We then converted the codes into numeric values (Supplementary Data Sheet 1).

Three preschool educators and three early STEM education researchers determined the validity of the two scientific exploration tasks and the coding scheme. They examined the tasks and rubrics as per the objectives of the study and age-appropriate requirements. Following the first validation, there was disagreement regarding some indicators and statements. For example, the *planning* indicator is coded as part of the inquiry capabilities and also as a self-regulation capability (Supplementary Data Sheet 1). Planning is a very important stage before engaging in any task or action and has many ways to be realized. Therefore, planning should be perceived as part of the scientific inquiry process and also when regulating cognition in this context and other contexts. All validators agreed that *planning* should be scored both in terms of inquiry skills and self-regulation. Disagreements were resolved by discussion until a consensus was reached, and only statements achieving full agreement were included in the analyses.

Three raters, specialists in early childhood science education research, coded 10% of the video data gathered in this study. The three raters watched the videos carefully (each video was viewed at least twice) and analyzed each video according to the coding scheme (Supplementary Data Sheet 1). Inter-rater reliability between the three raters was calculated, producing a Cronbach’s Alpha score ranging between 0.70 and 1 for the different indicators.

A fine-grained, video micro-analysis was conducted for each video (for a total of about 100 h of video recordings). Each video was carefully viewed, and every second was coded. We coded every statement the children made, as well as every gesture or facial expression.

Quantitative data were analyzed using the IBM SPSS Statistics software, version 25.0. Using this software, we calculated frequencies, mean values, *t*-tests for independent surveys, ANOVA, Bonferroni analysis, etc. Qualitative data included participants’ verbal responses, which were analyzed deductively, according to the indicators measured and the research questions. A mapping analysis was performed to identify relationships between the categories while reassigning them to the appropriate groups and arranging them by various indicators and on various levels (Strauss and Corbin, 1990; Shkedi, 2003). Finally, each indication was counted and added to the quantitative analysis.

## Results

The dependent variables in the current study were divided into nominal variables, ordinal variables, and variables on a numerical scale. Prior to examining the study questions and hypotheses, we examined whether the dependent variables measured numerically were normally distributed, using the Shapiro-Wilk tests. Due to the large variability in children’s ability at this age, some of the dependent variables were not normally distributed. Therefore, we examined the study questions and hypotheses by conducting parametric and non-parametric tests. The non-parametric analyses findings matched the findings of the parametric analyses. Therefore, we have presented the findings of the parametric analyses, which were measured on numerical scales.

### The Children’s Intuitive Inquiry Capabilities in an Open-Ended Task

The children’s inquiry capabilities were measured by five indicators: their ability to ask questions (total no. of questions), to use tools, to plan, to hypothesize, and to draw conclusions (see Supplementary Data Sheet 1). The means, standard deviations (SD), and the Min and Max values of their inquiry capabilities measures are presented in Table 1.

**TABLE 1:** Mean, SD, Min, and Max values of the children’s inquiry capabilities measure in an open-ended task (*N* = 215).

Children’s inquiry capabilities (range)	*M*	*SD*	*Min*	*Max*
Total no. of questions (number)	4.87	2.96	0.00	14.00
Competent use of tools (0–4)	2.07	1.16	0.00	4.00
Planning (0–7)	3.04	1.40	0.00	6.00
Hypothesizing (0–6)	2.14	1.39	0.00	5.00
Drawing conclusions (0–7)	4.32	1.81	0.00	7.00

As Table 1 indicates, there was high variability in the children’s inquiry capabilities. The highest variability was in the measure of the number of questions asked by the children during the entire scientific exploration. While ∼70% of the children asked between two and seven questions during the entire scientific exploration (146 children), a handful (10; i.e., 4.7%) asked over 10 questions. Therefore, the mean is 4.87 and the standard deviation is 2.96. The questions the participants asked were mostly of two types. The first type included questions related to visual aspects, such as: “What color is that?” or “How come it didn’t disappear?” The second type included questions related to activity, or instructions, such as: “Can I mix this?” or “Can I touch it?” We found that despite the children’s young age, and the fact that they did not have direct instruction or mediation on the part of the researcher, a few children succeeded in asking questions that testified to a high level of questioning, such as: “I mixed seltzer and oil. If I mix everything together, what will I get?”

Regarding the children’s ability to use tools, the mean was in the mid-range. Namely, only 13 children (13.0%) used all four tools competently. Regarding the children’s ability to plan and to hypothesize, none of the children attained the maximum score, and their abilities were average. Regarding the children’s ability to draw conclusions, their ability was in the mid-range. Only four children (1.9%) scored the maximum value of seven, available in this measure. However, this capability was manifested to the highest extent compared to competent tool use, planning, and hypothesizing.

### The Children’s Metacognitive Strategic Awareness and Self-Regulation Capabilities in an Open-Ended Task

The children’s metacognitive strategic awareness and self-regulation capabilities were measured by means of seven indicators: the level of the children’s strategic awareness, self-regulation, their lack of self-regulation, and their regulation of motivation: attention, persistence, autonomy, and engagement level) (see Supplementary Data Sheet 1). Table 2 presents the means, standard deviations, Min, and Max values of the children’s metacognitive strategic awareness and self-regulation capabilities measures.

**TABLE 2:** Mean, SD, Mdn, and range of the children’s metacognitive strategic awareness and self-regulation capabilities in an open-ended task (*N* = 215).

Children’s metacognitive and self-regulation capabilities (range)	*M*	*SD*	*Mdn*	*Min*	*Max*
Strategic awareness (0–4)	3.15	0.80	–	0.00	4.00
Self-regulation (0-sum of points)	10.47	3.11	–	2.00	19.00
Lack of self-regulation (0–7)	1.75	1.59	–	0.00	6.00
Attention^!^ (1–4)	3.53	0.61	4.00	2.00	4.00
Persistence^!^ (1–4)	3.15	0.75	3.00	1.00	4.00
Autonomy^!^ (1–4)	2.88	0.62	3.00	1.00	4.00
Engagement^!^ (1–4)	3.35	0.63	3.00	1.00	4.00

**^!^Variables are on ordinal scales; Means as well as Mdn are reported.**

As Table 2 shows, the children’s scores on the strategic awareness, attention, persistence, autonomy, and engagement measures were high, with a mean score of over 2.8 out of a maximum score of 4. Moreover, their scores on the lack of self-regulation measure were low, with a mean score of 1.75 out of a maximum score of 7. None of the participants scored the maximum score of 7 in the self-regulation measure, and only six children scored 6 on this measure (2.8%).

### Correlations Between the Children’s Inquiry Capabilities, Their Metacognitive Strategic Awareness, and Self-Regulation Capabilities in an Open-Ended Task

In order to examine whether correlations would be found between the children’s inquiry capabilities and their metacognitive strategic awareness, and self-regulation capabilities, Pearson and Spearman correlations analyses were conducted: Pearson correlations coefficients were calculated for the numerical scale variables, and Spearman correlations coefficients were calculated for the ordinal scale variables.

The analyses and calculation revealed significant positive correlations between the children’s five inquiry capabilities measures and the self-regulation capabilities measures. Specifically, we found the highest correlations between the children’s inquiry capabilities and their scores on the metacognitive strategic awareness, self-regulation, persistence, autonomy, and engagement measures. These results indicated that as the children’s scores on the metacognitive strategic awareness, self-regulation, persistence, autonomy, and engagement measures increased, their scores on the inquiry capabilities measures increased accordingly.

Note that although significant correlations were found among the 215 children who participated in the current study, their strengths were of a medium degree and below (all correlation coefficients were below 0.35). Moreover, no significant negative correlations were found between the five inquiry capabilities measures and their level of lack of self-regulation (see Table 3).

**TABLE 3:** Pearson and Spearman correlation coefficients between the children’s self-regulation capabilities and their ability to ask questions, to use scientific tools, to plan, to hypothesize, and to draw conclusions in the open-ended task (*N* = 215).

	Self-regulation capabilities						
Inquiry capabilities	Strategy awareness	Self-regulation	Lack of self-regulation	Attention^!^	Persistence^!^	Autonomy^!^	Engagement
Total number of questions	0.16*	0.24***	0.06	–0.10	0.11	–0.05	0.22***
Competent use of tools	0.32***	0.23*	–0.06	0.03	0.20**	0.14*	0.21**
Planning	0.20**	0.01	0.09	0.08	0.21**	0.13*	0.08
Hypothesizing	0.14*	0.05	0.07	0.11	0.17*	0.17*	0.08
Drawing conclusions	0.33***	0.16*	–0.10	0.15*	0.27***	0.25***	0.15*

*p < 0.05, **p < 01, ***p < 0.001. ^!^Variables are on ordinal scales; Spearman instead of Pearson correlation coefficients are reported.

Partial correlation analyses were conducted controlling for the general intelligence measured by the *Raven’s Progressive Matrices*, receptive vocabulary measured by the *Peabody Picture Vocabulary Test* and the age of the participants. The significant correlations remained significant and in some cases were even strengthened (see Supplementary Table 1).

### Correlations Between the Children’s Inquiry Capabilities and Their Metacognitive Strategic Awareness and Self-Regulation Capabilities in the Structured Task

In order to examine whether correlations would be found between the children’s inquiry capabilities and their metacognitive strategic awareness and self-regulation capabilities while conducting the structured task, Pearson and Spearman correlations analyses were conducted: Pearson correlations coefficients were calculated for the numerical scale variables, and Spearman correlations coefficients were calculated for the ordinal scale variables (Table 4).

**TABLE 4:** Pearson and Spearman correlation coefficients between the children’s self-regulation capabilities and their ability to ask questions, to use scientific tools, to plan, to hypothesize, and to draw conclusions in the structured task (*N* = 147).

	Self-regulation capabilities						
Inquiry capabilities	Strategy awareness	Self-regulation	Lack of self-regulation	Attention^!^	Persistence^!^	Autonomy^!^	Engagement
Total number of questions	0.10	0.32***	0.16	–0.12	−0.33***	−0.38***	0.20*
Competent use of tools	0.38***	0	0.30***	−0.23**	–0.12	–0.15	0.19*
Planning	0.14	0.08	0.14	–0.06	0.10	0.04	–0.04
Hypothesizing	0.12	0.10	0.11	–0.06	0.11	–0.07	0.29***
Drawing conclusions	0.34***	0.20*	0.03	0.05	–0.01	–0.04	0.08

*p < 0.05, **p < 01, ***p < 0.001. ^!^Variables are on ordinal scales; Spearman instead of Pearson correlation coefficients are reported.

The analyses and calculation revealed significant positive correlations between four of the five children’s inquiry capabilities measures and the self-regulation capabilities measures. Specifically, we found correlations between the total number of questions asked, competent use of tools, hypothesizing, and drawing conclusions and their scores on self-regulation. Negative significant correlations were found with the variables of attention (with competent use of tools), persistence, and autonomy (with total number of questions asked). These findings will be further elaborated in the “Discussion” section.

Partial correlation analyses were conducted controlling for the general intelligence measured by the *Raven’s Progressive Matrices*, receptive vocabulary measured by the *Peabody Picture Vocabulary Test* and the age of the participants. The significant correlations remained significant and in some cases were even strengthened (see Supplementary Table 2).

### Differences in the Children’s Intuitive Inquiry, Metacognitive Strategic Awareness, and Self-Regulation Capabilities by Type of Task

In the current study, 147 children engaged in a structured task in addition to the open-ended task. In order to examine the differences between the two types of tasks in the dependent variables, we performed three different analyses, depending upon the variable scale. To examine the differences between the two tasks on the ordinal scale variables, we performed Wilcoxon analyses; and to examine the differences between the two tasks on the numerical scale variables, we performed paired samples *t*-test analyses.

#### Differences in the Children’s Inquiry Capabilities by Type of Task

We found significant differences between the two task types in the number of questions the children asked during the entire scientific exploration process, their ability to use tools, and their ability to plan. Table 4 presents the means and SD of the children’s *inquiry capabilities* measures by type of task.

As Table 5 shows, the number of questions asked by the children during the scientific exploration tasks and their ability to plan were significantly higher in the structured task than in the open-ended task. However, the children’s ability to use tools competently was significantly higher in the open-ended task than in the structured task.

**TABLE 5:** Mean, SD, and *t*-values of the scores of the children’s inquiry capabilities measures by type of task (*n* = 147).

	Open-ended		Structured				
Inquiry capabilities measures	*M*	*SD*	*M*	*SD*	*t*	*P*	Cohen’s *d*
Total number of questions (number)	5.04	3.00	5.78	4.62	−2.16*	0.032	0.18
Competent use of tools (0–4)	2.20	1.20	1.57	1.20	5.56***	0.000	0.46
Planning (0–7)	3.01	1.46	3.83	1.48	−5.25***	0.000	0.43
Hypothesizing (0–6)	1.94	1.48	2.01	1.49	–0.47	0.640	0.04
Drawing conclusions (0–7)	4.14	1.91	4.37	1.53	–1.27	0.207	0.10

*p < 0.05; ***p < 0.001.

#### Differences in the Children’s Metacognitive Strategic Awareness and Self-Regulation Capabilities by Type of Task

Significant differences were found between the two task types in the scores on self-regulation, lack of self-regulation, attention, persistence, and autonomy. Table 6 presents the means and SD of the children’s *metacognitive strategic awareness and self-regulation* measures by task type.

**TABLE 6:** Mean, SD, *t*- and *z*-values of the children’s metacognitive strategic awareness and self-regulation capabilities by task type (*N* = 147).

	Open-ended			Structured				
Children’s self-regulation capabilities	*M*	*SD*	*Mdn*	*M*	*SD*	*Mdn*	*t/Z*	*p*
Strategic awareness	3.15	0.88	–	3.06	0.72	–	1.12	0.265
Self-regulation	10.77	2.84	–	7.99	3.14	–	9.54***	0.000
Lack of self-regulation	1.80	1.66	–	1.33	1.63	–	2.99**	0.003
Attention^!^	3.46	0.63	4.00	3.63	0.60	4.00	2.98**	0.003
Persistence^!^	3.14	0.79	3.00	3.51	0.64	4.00	4.70***	0.000
Autonomy^!^	2.86	0.70	3.00	3.18	0.65	3.00	4.72***	0.000
Engagement^!^	3.33	0.66	3.00	3.37	0.49	3.00	0.86	0.391

**p < 0.01; ***p < 0.001. ^!^Variables are on ordinal scales; Means and Mdn are reported.

As Table 6 shows, the scores on the self-regulation and lack of self-regulation measures were significantly higher in the open-ended exploration task as compared to the structured exploration task. During the open-ended task, participants showed significantly higher frequency and level of evidences-manifesting checking and monitoring, controlling, and evaluating (Supplementary Data Sheet 1). The scores on the attention, persistence, and autonomy measures were significantly higher in the structured task than in the open-ended task. These differences will be elaborated in the “Discussion” section.

## Discussion and Conclusion

This study sought to examine nascent inquiry, metacognitive strategic awareness, and self-regulation capabilities among preschoolers during play-based scientific exploration tasks (open-ended, and structured). In addition, it sought to examine the relationship between inquiry and self-regulation capabilities while preschoolers engage in play-based scientific exploration tasks.

### Inquiry Capabilities During the Open-Ended Exploration Task

The study findings show that given the opportunity, children quite naturally exhibit inquiry capabilities during situated scientific exploration: they ask questions, plan, hypothesize, use tools, draw conclusions, and can explain their conclusions. This study’s findings are in line with findings of other studies indicating that young children hypothesize and can modify hypotheses when necessary (Legare, 2012); pose scientific queries; comprehend basic scientific concepts (Patrick et al., 2009); and use hypotheses to predict results, evaluate evidence, and produce explanations (Piekny et al., 2014; Walker et al., 2014). Moreover, children’s free play involves intuitive experiments that enable them to activate complex mechanisms and comprehend phenomena better (Gopnik, 2012). Most of the studies so far have not tested these capabilities in young children in the context of ecologically situated scientific explorations. The current study is innovative in its focus on children’s inquiry capabilities in contexts of authentic, play-based scientific exploration. This study reveals that when presented with materials and equipment of a scientific nature, even without setting explicit goals and rules, children do exhibit scientific inquiry capabilities.

The results suggest that the children’s ability to draw conclusions was manifested to a greater extent than other inquiry capabilities tested, given the number of children who succeeded in drawing conclusions without mediation, as well as the level of their conclusions. Drawing conclusions is the final stage of the inquiry process, leading to the production of new information and posing new research questions. ErgaZaki and ZogZa (2013) reason that involvement in inquiry improves inductive ability. The findings that have emerged in our study are in line with that claim. The children in our study referred to what happened to the materials during their explorative actions. For example, when they mixed water with oil, they could see that the oil floated on the water, but when they mixed juice with milk, the juice tinted the milk and changed its color. Therefore, in accordance with the concrete, visible results, the children were able to draw conclusions. Other inquiry capabilities, such as planning and hypothesizing, involve more complex, abstract thinking skills that are of a predictive type, rather than being based on concrete, visual information. Thus, capabilities such as planning and hypothesizing are more challenging, as they are based on abstract thought and thinking a few steps ahead.

Scholarly consensus holds that the skill of posing questions is fundamental to inquiry processes and contributes to cognitive development and higher-order thinking (National Research Council (NRC), 2012). In terms of the nature of science, it is important that children understand that with the aid of questions, they access new information they did not previously have (Kuhn and Dean, 2005). In our study, the children’s ability to pose questions developed during the course of the open-ended exploration task. The participants actively explored the use of tools and materials, and observed what happened to the materials after mixing them, which generated more questions about the task, such as how the tools work, etc.

Engaging in science is a complex process that leads to creating new knowledge. Zimmerman and Croker (2013) contend that knowledge of science and mastery of inquiry skills are mutually enriching and lead to the development of scientific thinking. This bears out the importance of providing children with play-based scientific exploration experiences that include exposure to a range of phenomena, tools, and materials that, with time, become familiar, enrich their knowledge of science, and raise the level of their inquiry capabilities (Tunnicliffe, 2015).

### Metacognitive Strategic Awareness and Self-Regulation Capabilities During the Open-Ended Exploration Task

The second research question sought to study nascent metacognitive strategic awareness and self-regulation capabilities among children during scientific exploration tasks. The study examined four parameters (comprising 13 indicators; Supplementary Data Sheet 1) that characterize the emerging metacognitive strategic awareness and self-regulation capabilities in children. According to our findings, the young participants exhibited capabilities of metacognitive strategic awareness, self-regulation, and a very high level of attention, persistence on task, and engagement. They exhibited abilities of monitoring and control, such as defining difficulty and then addressing a solution; they exhibited capabilities of testing and evaluating; they expressed satisfaction or dissatisfaction with the results and could explain why.

This study corroborates the results of other studies, indicating that through the use of age-appropriate methodology, such as real-time (online) data gathering while young children are engaged with a task, we can see the nascent abilities of planning, monitoring, control, and reflection in young children (Whitebread and Coltman, 2010; Whitebread et al., 2010; Bryce and Whitebread, 2012). Our results are in line with studies showing that preschool-aged children can plan, set goals, and conduct reflective processes on their learning (Whitebread et al., 2007; Shamir et al., 2009; Larkin, 2010; Whitebread and Coltman, 2010).

The literature indicates that children activate strategies when their task is appropriate and of significance for them (Whitebread and Coltman, 2010). In our study, despite the children’s young age, and the fact that most of them experienced scientific exploration of this type for the first time, one conspicuous result was their low score on the measure of lack of self-regulation. The average lack of self-regulation score was 1.8 (out of a maximum of 7). Consequently, the researchers could only observe indications such as brute force and repeated errors in rare instances.

Self-regulation includes the ability to control the intensity of one’s responses. Metacognitive experiences relate to the emotional aspect of learning, including motivation and social-emotional processes (Efklides, 2006). These occur in the learner through cognitive experiences, including feelings, judgments, and knowledge about the task. Self-regulation is therefore one of the manifestations of reciprocity between emotion and cognition (Lee, 2005). In this study, we examined four measures related to emotion and motivation: attention, persistence, autonomy, and engagement with the task.

Most of the children worked happily while maintaining their focus on the task. They persevered independently while facing the difficulties that arose during the task, and exhibited involvement, interest, and enjoyment during its performance. When children engage in age- and developmental stage-appropriate activity, they perform better in aspects like self-regulation and cognitive procedures, as well as emotionally and behaviorally (Efklides, 2006).

Consistent with the literature (e.g., Bronson, 2000; Winne and Perry, 2000; Winne, 2018), this study shows that even without formal instruction in the subject area, without explicit task instructions, and without explicit goals, preschoolers succeeded in implementing components of scientific inquiry and exhibiting manifestations of metacognitive strategic awareness and self-regulation during an open-ended scientific exploration task that included the use of tools and materials.

### Relationship Between Inquiry, Metacognitive Strategic Awareness, and Self-Regulation Capabilities During an Open-Ended Exploration Task

The study results support our initial hypothesis and indicate significant correlations between the five measures of preschoolers’ inquiry capabilities and manifestations of metacognitive strategic awareness and self-regulation. Specifically, we found correlations between the children’s inquiry capabilities and most of their scores on the strategic awareness, self-regulation, persistence, and engagement measures in the open-ended play-based task. These results indicated that as the children’s scores on the inquiry capabilities measures increased, the scores on the metacognitive strategic awareness, self-regulation, persistence, and engagement measures increased accordingly. As the children’s strategic awareness and self-regulation grew while they were performing the task, skills such as planning the process, gathering data, making the connection between cause and effect, and drawing conclusions improved. Through using these skills, they succeeded in addressing the questions themselves: “What is the nature of this task?,” “What type of strategies do I need to adopt in order to succeed?,” “Did I choose the right strategy or shall I change it?,” and so forth.

Data analysis revealed no significant correlations between the five inquiry capabilities measures and the children’s level of lack of self-regulation. As previously mentioned, the result shows that even without formal instruction of scientific inquiry skills, preschoolers succeeded in monitoring and controlling their actions and responses, while rarely showing brut or negative responses, nor repeated errors.

Note that among the 215 children who participated in the current study, the correlations between the five inquiry capabilities measures and manifestations of metacognitive strategic awareness and self-regulation, were significant but weak. These results might be explained by virtue of the very young age of the participants of this study, whose thinking and learning capabilities were still in the early stages of development. The variability in their inquiry level of performances (SD presented in Table 1) might explain the weak correlations between the measures tested. The significant correlations, although weak, support our hypothesis, yet further studies are needed with a possible refinement of the coding scheme and of the play-based exploration task itself.

This study corroborates studies that attest to significant correlations between inquiry skills and self-regulation and metacognitive thinking. The defining feature of scientific thinking is the set of cognitive and metacognitive skills involved in differentiating and coordinating theory and evidence. In particular, it is precisely metacognitive awareness that differentiates more from less sophisticated scientific thinking (Kuhn, 2002). Scientific thinking is an umbrella term that encompasses the reasoning and problem-solving skills used in generating, testing, and revising hypotheses or theories, and in the case of fully developed skills, reflecting on the process of knowledge acquisition and modification. Acquired inquiry skills lead to knowledge modification, and in turn, this developing knowledge influences the development of more sophisticated inquiry skills. Concurrently, as metacognitive capabilities develop, children and adolescents gain a better understanding of the nature of inquiry and the use of skills (Zimmerman, 2007). For these reasons, examining the relations between inquiry, metacognitive strategic awareness, and self-regulation capabilities during an open-ended, play-based exploration task, bear great importance toward developing appropriate early science pedagogy.

### Inquiry, Metacognitive Strategic Awareness, and Self-Regulation Capabilities During Structured Scientific Exploration Task

This study has attempted to examine nascent inquiry, metacognitive strategic awareness, and self-regulation capabilities in preschoolers during a structured exploration task and compare them to the same abilities exhibited during an open-ended exploration task. Our motivation was to examine whether preschoolers’ capabilities will differ in the context of structured task which is aligned with the view that young children need guidance and explicit instruction to acquire the components of scientific inquiry (Zimmerman, 2007) compared to the context of open-ended, play-based task–allowing the children to apply and test their intuitive theories and improve their nascent inquiry capabilities (Buchsbaum et al., 2011).

The study findings indicate significant differences between the two task types regarding the children’s capabilities to plan, use tools, and pose questions. While each of the scientific tasks – structured and open-ended – both invite and emphasize inquiry capabilities at various intensities among the study participants, the ability to plan was better manifested during the structured task.

It is likely that when the task establishes a final goal, it is easier for children to plan their steps toward reaching it. The ability to use tools was better manifested in the open-ended, play-based exploration task. The open-ended task provided the participants with endless options to make attempts and to carry out the experiments with the tools and materials that were situated in front of them. In the structured exploration task, however, the children were focused on the instructions and the actions required for carrying out the task, and they did not necessarily need to undertake many actions or use many tools in order to do so. The number of questions asked during the exploration process was greater in the structured task; however, during both tasks, questions arose regarding the use of tools and materials and about how to carry out the task. This finding is in line with Samarapungavan et al. (2008) who affirmed that questioning encourages reflective scientific thinking and learning.

Our findings show that although the children manifested higher levels of attention, persistence, and autonomy during the structured task, they manifested lower levels of self-regulation. These findings may indicate that the young participants could better regulate their emotions (while, to a lesser degree, regulating their cognition) when they had clear task instructions. Considering that the cognitive and emotional aspects of self-regulation can be correlated (Efklides, 2006), these findings may appear to be contradictory; however, they are not: The more demanding task of the two was the structured exploration task, which involved specific instructions and required more self-regulation than the open-ended task. Despite the particularly young age of the participants, the clear instructions provided for the structured exploration task reduced their emotional load, thus enabling them to muster the effort needed to accomplish the task.

However, during both tasks, the children were active during the exploration. This likely stems from the fact that the open-ended task enabled the children to make their own decisions on the matter of what to investigate with the items at hand, while the structured task included clear instructions with required, defined results.

Data analysis revealed weak but significant correlation coefficients between the children’s self-regulation capabilities and some inquiry capabilities in the structured task and in the open-ended task. These significant correlations remained after controlling for age, PPVT, and Raven scores. These findings show that both crystalized and fluid intelligence positively support regulation of cognition and actual scientific exploration capabilities. Intelligence is associated with a greater ability to regulate one’s impulses, emotions, and behavior, and may further explain why these two traits (intelligence and self-control) are generally so closely related to important life outcomes such as success in primary and secondary education. A growing body of convincing evidence suggests that intelligence is closely tied to the development of self-regulation in the early stages of the course of life (Primi et al., 2010).

The literature addresses the effects of structured environments vs. those of open environments on engagement in science and exploratory processes among children (Chinn and Malhotra, 2002; Bergen, 2009). Most studies recommend providing a structured environment, based on research showing that without guidance and explicit instructions, children find it difficult to acquire the components of scientific thinking, as well as to implement scientific thinking skills such as planning experiments, documentation, and processing existing knowledge with new knowledge. In other words, they should be taught these processes in a structured manner (Zimmerman, 2007). According to this view, a curriculum that emphasizes a structured planning of science engagement – including details regarding the type of activity, the content, and the sequence – mitigates the cognitive burden and maintains the focus on the goals. Such an approach even improves children’s cognitive, lingual, and emotional level of engagement in science (Weisberg et al., 2013). The results of the present study do support this approach. However, although the participants in this study showed better inquiry capabilities in the structured scientific exploration task, this was not the case regarding self-regulation capabilities. Significant higher scores of self-regulation manifestations were revealed in the context of the open-ended, play-based, exploration task. Other researchers also support open-ended exploration, based on studies attesting to the effectiveness of learning that stems from spontaneous investigation, as well as testimonies that this method improves the young students’ cognitive, emotional, and social abilities (Bergen, 2002; Hyson, 2003), as well as their creative imagination. The results of the present study suggest the importance of combining various learning environments and experiences in science instruction that encourage children to engage in structured exploration alongside spontaneous, play-based, open-ended, exploration.

### Limitations of the Study

Working with 5- to 6-year-old children requires adapting the research tools to meet the cognitive, linguistic, emotional, and behavioral capabilities of children at this age. Thus, due to the age limitations of the research sample, the findings are based primarily on deriving meaning from observing the children’s behaviors, rather than being solely based upon what a child reported verbally. Although the validation process of the tasks and coding scheme was accomplished with six experts, and the inter-rater reliability was accomplished with three experts who coded 10% of the video data, for the sake of reliability, future studies should analyze a greater percentage of the video data, given the relatively high inference used in the coding system.

The exploration tasks and coding scheme of this study were developed especially for preschool-age children and were based on the children’s behavioral and verbal responses. However, the tasks lack direct questions addressed to the children, seeking to find out what they think about structured vs. open-ended exploration tasks. Future research should address this gap.

Regarding the study procedure, although the open-ended and structured tasks were presented in a counterbalanced manner, one may argue that those children who began with the structured task may have experienced behaviors associated with structure and clear goals, leading to higher incidences of inquiry skills, metacognitive strategic awareness, and self-regulation. In turn, this may have affected their performance of the open-ended task. This issue can be investigated by further data analysis. Findings may have potential pedagogical implications and can enrich the discourse on children’s intuitive capabilities and the ways they develop.

In this study, the children’s experiences during the open-ended exploration task enabled them to decide what to explore using the tools and materials at their disposal. While they decided how to act, they were limited in terms of location; in other words, they worked at a table, over a tray, and could not choose to work elsewhere. Future research should examine these abilities through an open-ended task that allows the children to decide what, how, and where (for example, outside in the schoolyard) to explore. This would offer researchers the opportunity to provide the children with additional phenomena to investigate.

Another limitation of this study may lie with the measurements of each variable in the scoring scheme. For example, some behavioral items in the scoring scheme of scientific inquiry skills overlapped with those of metacognitive strategic awareness and self-regulation. Specifically, *planning* appears in the metacognitive variables and also as a variable of the inquiry capabilities. Each *planning* variable refers to a different issue: in one instance it is the planning of physical actions, while the other refers to planning on a mental level (e.g., how to monitor my strategies).

We considered this potential bias when developing the coding scheme. For that reason, we conducted an intensive validation process, with six preschool educators and early STEM education researchers. However, more accuracy is needed to differentiate between these variables. These overlaps pose a potential risk of overestimating correlations among the variables and deviating from the study assumptions. Thus, further studies are needed to refine these measures.

## Summary

To conclude, this study offers insights related to various aspects of nascent scientific thinking in children, focusing on inquiry, metacognitive strategic awareness, and self-regulation capabilities. Few studies have examined these nascent abilities in children while performing situated, scientific exploration of materials. This study shows that preschoolers like to explore and engage in science activities, and can maintain their attention, persistence, and engagement. Preschoolers exhibit verbal and behavioral responses and actions that demonstrate nascent inquiry capabilities, as well as indications of metacognitive strategic awareness and self-regulation capabilities. Some of these capabilities are better manifested during structured exploration, and others during open-ended exploration tasks. Significant correlations were found between these capabilities. These findings indicate the importance of offering children a learning environment that provides them with rich opportunities to explore and develop their intuitive inquiry capabilities: An environment that draws children into active involvement in the inquiry process and development of nascent metacognitive awareness and self-regulation capabilities.

## Data Availability Statement

The datasets generated for this study are available on request to the corresponding author.

## Ethics Statement

The studies involving human participants were reviewed and approved by Rene Osizon, Ethics Committee (#10495), The Office of the Chief Scientist of the Ministry of Education. Written informed consent to participate in this study was provided by the participants’ legal guardian/next of kin.

## Author Contributions

All authors contributed by editing the literature review, collecting the data, analyzing the data, drawing the conclusions, and writing and editing the manuscript.

## Conflict of Interest

The authors declare that the research was conducted in the absence of any commercial or financial relationships that could be construed as a potential conflict of interest.
