# Supplementary material for: Nascent Inquiry, Metacognitive, and Self-Regulation Capabilities Among Preschoolers During Scientific Exploration
Source: Front Psychol. 2020 Jul 21;11:1790. doi: 10.3389/fpsyg.2020.01790 (PMC7396714; doi:10.3389/fpsyg.2020.01790)
Supplement: Supplementary file 1 [file Data_Sheet_1.docx]

**Appendices**

**Appendix 1A — Scoring scheme of preschoolers' scientific inquiry skills**

| **Inquiry skills indicators** | **Variables and examples** | **Range** |
| --- | --- | --- |
| Planning | Planning before the task:  Child planned spontaneously  Child planned after one mediation  Child planned after two mediations | 1  0-1  0-1  0-1 |
|  | Planning levels:  None  Irrelevant planning (e.g., "I'll build a house with them or paint.")  One-step planning—The child only refers to mixing the materials (e.g., “I’m gonna mix the oil and the water to see what happens.”).  Dual-step planning—The child refers to the materials and the use of tools or the order of action (e.g., “I can fill the spoon with juice and then pour it into the milk and mix it, and that way it’ll be another color.”)  Multiple-step planning—The child refers to the order of operations, tools, and materials (e.g., “I’m gonna pour some of the milk into the juice and then I’m gonna mix it with the spoon. And then I’m gonna fill the dropper with soda and make it drip slowly. And that way there’s gonna be an explosion.” | 0-4 |
|  | **Planning—Total score** | 0-7 |
| Hypothesizing | Phrasing hypothesis:  Spontaneously  After mediation | 0-1  0-1 |
|  | Level of hypothesis:  None  Irrelevant hypothesis (e.g., "I'll make a beautiful drawing.")  Descriptive hypothesis (e.g., "The water will become red.")  Hypothesis that indicates comprehension (e.g., "The oil will float on water.")  Hypothesis that indicates application (e.g., "The numbers on the syringe are like the numbers on the measuring spoon for my medicine at home. So, the water will come up to here.") | 0-4 |
|  | **Ability to hypothesize—Total score** | 0-6 |
| Competent use of scientific tools | Use of syringe (no/yes)  Use of funnel (no/yes)  Use of dropper (no/yes)  Use of a measuring spoon (no/yes) | 0-1  0-1  0-1  0-1 |
|  | **Competent use of scientific tools—Total score** | 0-4 |
| Drawing conclusions | Draw conclusions—spontaneously  Draw conclusions—after mediation  Draw conclusions—justified the answer | 0-1  0-1  0-1 |
|  | Level of conclusion:  None  Irrelevant conclusion (e.g., "It turned out to be an animal.")  Descriptive conclusion (e.g., "It changed colors because I mixed them all.")  A conclusion that indicates a process of understanding (e.g., "The mixture came out pink because I used a dropper, so there were only a few drops added to the milk.")  Prior knowledge and understanding the procedure (e.g., "The oil is on top of the water. I knew this was going to happen because I saw it on a TV show.") | 0-4 |
|  | **Drawing conclusions—Total score** | 0-7 |
| Questioning | No questions  Asking spontaneous questions before/during/after the scientific experiment (3 points each)  Asking questions after the first mediation before/after the task (2 points each)  Asking questions after the second mediation before/after the task (1 point each) | 0  0-9  0-4  0-2 |
|  | **Questioning—Total score** | 0-15 |
| Number of questions | Total questions asked during the entire procedure | 1 point per question |
| Level of questions | Score was determined according the highest level the child reached:  Level 0 – No questions  Level 1 – Irrelevant questions (e.g., “Do you know my mother?”)  Level 2 – Descriptive Questions (can be answered using the senses or by asking permission to do something; e.g., “What color is the oil?”)  Level 3 – Comprehension questions (What? How much? Why? How? Where? (e.g., “How come there’s no milk here?”)  Level 4 – Application Questions – Apply previous knowledge; relate to other contexts; based on existing knowledge and skills to apply in another situation; comparison questions (e.g., “Does the syringe have a small hole because we want the liquid to run out slowly?”)  Level 5 – Questions that indicate systemic thinking; consuming research and problem-solving to obtain an answer (analysis, synthesis, inclusion; e.g., What happens if I pour the pink liquid on the ground? Will it make everything grow pink? Or will there be brown and red?”) | 0-5 |

**Appendix 1B — Scoring scheme of preschoolers' metacognitive and self-regulation capabilities**

| **Metacognition and self-regulation** | **Indicators** | **Variables and examples** | **Range** |
| --- | --- | --- | --- |
| Metacognitive awareness – structured, task only | Metacognitive strategic awareness | Planning before starting (no/yes)  Looking at the tools and materials several times during the experience (no/yes)  Selecting/focusing on relevant tools and materials (e.g., chooses a cup when s/he wants to add a large amount of liquid and chooses a dropper to add a small amount of liquid; no/yes)  Pausing to think (no/yes) | 0- 1  0-1  0-1  0-1 |
|  |  | **Strategic awareness — Total score** | 0-4 |
| Self-regulation | Task awareness | No indication  Child shows indications of being aware of previous similar tasks or understanding the current task (e.g., "I have to mix the oil with water"; "I can get the color red from here"). | 0  1 point per each instance/indication |
|  | Planning | No indication  The child makes a reference or plans future actions pertinent to the task (e.g., arranges the space, materials, and tools / "I'll pour it here and then mix it.") | 0  1 point per each instance/indication |
|  | Monitoring & debugging | Checking:  Child regularly checks the plan or what happens to the materials while experimenting: No indication, one indication, two indications, three or more indications. | 0-3 |
|  | Evaluating | The child verbally assesses the difficulty of the task. Verbal responses (e.g., "It's hard"; "I can't pull the syringe!") and non-verbal responses (e.g., looking proud or frustrated with the task). | 1 point per instance/indication |
|  | Controlling | Changing strategy:  The child changes strategy after having noticed an error.  The child changes the way of handling materials or tools.  No indication, one indication, two indications, three or more indications.  Pauses to think (i.e., about the exploration process or when facing difficulty). | 0-3  1 point per each instance/indication |
|  |  | **Self-Regulation — Total score** | **0-sum of points** |
| Lack of self-regulation | Brute force | The child persistently tries to exert excessive force on tools; The child makes a great mess with the materials; demonstrates impulsivity.  No indication, one indication, two indications, three or more indications. | 0-3 |
|  | Off task | The child completely disengages from the task to engage in unrelated behavior (no/yes) | 0-1 |
|  | Repetition of error | The child repeatedly makes the same mistake, with no change in strategy (e.g., uses a funnel upside down; uses a syringe to collect powder). Persist on error without continuing with the task. | 0-3 |
|  |  | **Lack of self-regulation — Total score** | **0-7** |
| Emotional and motivational  Regulation | Attention | Child is occupied in irrelevant conversation or activity/ often distracted/ distracted, but can return to task/sustains attention and maintains focus on task | 1-4 |
|  | Persistence | Gives up on the task / continuous unstable persistence/ disrupted persistence / continuous persistence | 1-4 |
|  | Autonomy | Child is very dependent /often asks for help/ occasionally asks for help/ works independently | 1-4 |
|  | Engagement | Visible signs of boredom or an unwillingness to engage/ the child loses interest quickly / maintains an effort / shows signs of pleasure | 1-4 |
|  |  |  |  |
