# Supplementary material for: Nascent Inquiry, Metacognitive, and Self-Regulation Capabilities Among Preschoolers During Scientific Exploration
Source: Front Psychol. 2020 Jul 21;11:1790. doi: 10.3389/fpsyg.2020.01790 (PMC7396714; doi:10.3389/fpsyg.2020.01790)
Supplement: Supplementary file 2 [file Data_Sheet_2.docx]

**Supplement**

Supplementary Table 1

*Partial correlation coefficients between the children’s self-regulation capabilities and their ability to ask questions, to use scientific tools, to plan, to hypothesize, and to draw conclusions in the open-ended task controlling for age, PPVT and Raven scores (N = 215).*

|  | Self-regulation capabilities | | | | | | |
| --- | --- | --- | --- | --- | --- | --- | --- |
| Inquiry capabilities | Strategy awareness | Self-regulation | Lack of self-regulation | Attention^!^ | Persistence^!^ | Autonomy^!^ | Engagement |
| Total number of questions | 0.16* | 0.31*** | 0.07 | -0.15* | 0.06 | -0.11 | 0.22*** |
| Competent use of tools | 0.30*** | 0.21** | -0.06 | 0.05 | 0.21** | 0.17* | 0.23*** |
| Planning | 0.19** | 0.00 | 0.08 | 0.08 | 0.18** | 0.16* | 0.11 |
| Hypothesizing | 0.14* | 0.03 | 0.06 | 0.11 | 0.18** | 0.17* | 0.11 |
| Drawing conclusions | 0.33*** | 0.14* | -0.11 | 0.16* | 0.31*** | 0.29*** | 0.21** |

****p* < 0.05, ***p* < 01, ****p* < 0.001; ^!^ variables are on ordinal scales; Spearman instead of Pearson correlation coefficients are reported.**

Supplementary Table 2

*Partial correlation coefficients between the children’s self-regulation capabilities and their ability to ask questions, to use scientific tools, to plan, to hypothesize, and to draw conclusions in the structured task controlling for age, PPVT and Raven scores (N=147)*

|  | Self-regulation capabilities | | | | | | |
| --- | --- | --- | --- | --- | --- | --- | --- |
| Inquiry capabilities | Strategy awareness | Self-regulation | Lack of self-regulation | Attention^!^ | Persistence^!^ | Autonomy^!^ | Engagement |
| Total number of questions | 0.07 | 0.25** | 0.11 | -0.13 | -0.26*** | -0.43*** | 0.22** |
| Competent use of tools | 0.35*** | 0.47*** | 0.28*** | -0.22** | -0.13 | -0.14 | 0.18* |
| Planning | 0.12 | 0.04 | 0.12 | -0.05 | 0.05 | 0.09 | -0.06 |
| Hypothesizing | 0.09 | 0.08 | 0.10 | -0.02 | 0.08 | -0.12 | 0.29*** |
| Drawing conclusions | 0.33*** | 0.20* | 0.03 | 0.23** | 0.10 | -0.01 | 0.02 |

****p* < 0.05, ***p* < 01, ****p* < 0.001; ^!^ variables are on ordinal scales; Spearman instead of Pearson correlation coefficients are reported.**
